# Single Crystalline Films of Ce^3+^-Doped Y_3_Mg_x_Si_y_Al_5−x−y_O_12_ Garnets: Crystallization, Optical, and Photocurrent Properties

**DOI:** 10.3390/ma16051869

**Published:** 2023-02-24

**Authors:** Vitaliy Gorbenko, Tetiana Zorenko, Anna Shakhno, Paweł Popielarski, Andres Osvet, Miroslaw Batentschuk, Alexander Fedorov, Sebastian Mahlik, Tadeusz Leśniewski, Natalia Majewska, Yuriy Zorenko

**Affiliations:** 1 Institute of Physics, Kazimierz Wielki University in Bydgoszcz, 85-090 Bydgoszcz, Poland; 2 Institute of Materials for Electronics and Energy Technology (i-MEET), Department of Materials Science and Engineering VI, University of Erlangen-Nürnberg, 91058 Erlangen, Germany; 3 SSI Institute for Single Crystals, National Academy of Sciences of Ukraine, 61178 Kharkiv, Ukraine; 4 Institute of Experimental Physics, Faculty of Mathematics, Physics and Informatics, University of Gdansk, Wita Stwosza 57, 80-308 Gdansk, Poland

**Keywords:** luminescence, single crystalline films, liquid-phase epitaxy, Mg^2+^–Si^4+^-based garnet, Ce^3+^-doped, phosphor converters, photocurrent, scintillators

## Abstract

This research focuses on LPE growth, and the examination of the optical and photovoltaic properties of single crystalline film (SCF) phosphors based on Ce^3+^-doped Y_3_Mg_x_Si_y_Al_5−x−y_O_12_ garnets with Mg and Si contents in x = 0–0.345 and y = 0–0.31 ranges. The absorbance, luminescence, scintillation, and photocurrent properties of Y_3_Mg_x_Si_y_Al_5−x−y_O_12_:Ce SCFs were examined in comparison with Y_3_Al_5_O_12_:Ce (YAG:Ce) counterpart. Especially prepared YAG:Ce SCFs with a low (x, y < 0.1) concentration of Mg^2+^ and Mg^2+^–Si^4+^ codopants also showed a photocurrent that increased with rising Mg^2+^ and Si^4+^ concentrations. Mg^2+^ excess was systematically present in as-grown Y_3_Mg_x_Si_y_Al_5−x−y_O_12_:Ce SCFs. The as-grown SCFs of these garnets under the excitation of α–particles had a low light yield (LY) and a fast scintillation response with a decay time in the ns range due to producing the Ce^4+^ ions as compensators for the Mg^2+^ excess. The Ce^4+^ dopant recharged to the Ce^3+^ state after SCF annealing at T > 1000 °C in a reducing atmosphere (95%N_2_ + 5%H_2_). Annealed SCF samples exhibited an LY of around 42% and similar scintillation decay kinetics to those of the YAG:Ce SCF counterpart. The photoluminescence studies of Y_3_Mg_x_Si_y_Al_5−x−y_O_12_:Ce SCFs provide evidence for Ce^3+^ multicenter formation and the presence of an energy transfer between various Ce^3+^ multicenters. The Ce^3+^ multicenters possessed variable crystal field strengths in the nonequivalent dodecahedral sites of the garnet host due to the substitution of the octahedral positions by Mg^2+^ and the tetrahedral positions by Si^4+^. In comparison with YAG:Ce SCF, the Ce^3+^ luminescence spectra of Y_3_Mg_x_Si_y_Al_5−x−y_O_12_:Ce SCFs greatly expanded in the red region. Using these beneficial trends of changes in the optical and photocurrent properties of Y_3_Mg_x_Si_y_Al_5−x−y_O_12_:Ce garnets as a result of Mg^2+^ and Si^4+^ alloying, a new generation of SCF converters for white LEDs, photovoltaics, and scintillators could be developed.

## 1. Introduction

White light-emitting diodes (WLEDs) are quickly displacing conventional sources of light due to their high energy efficiency, extended exportation time, high luminous efficiency, and environmental friendliness [1]. These devices, including yellow-emitting Y_3_Al_5_O_12_:Ce (YAG:Ce) garnet powders and blue LED chips, are regarded as standard WLED light sources [2]. High-power WLEDs can be produced using YAG:Ce crystals, translucent ceramic phosphors [3,4,5], eutectics [6], and epitaxial structures [7,8] combined with blue LEDs. As a result of the garnet structure’s high degree of flexibility, which permits various cation substitutions in the octahedral {A}, dodecahedral [B] and tetrahedral (C) sites, it is possible to alter the composition of {Y}_3_[Al]_2_(Al)_3_O_12_ garnets in order to optimize its Ce^3+^ spectroscopic properties and fulfill the demand of WLED applications. For the development of high-power white LEDs, a new class of garnet phosphors based on Ce^3+^-doped A_3_B_2_C_3_O_12_ ({A} = Ca, Y and rare earth ions; [B] = Sc, Ga, Mg, Al; (C) = Al, Si, Ga) garnets were also proposed [9,10,11,12,13,14,15,16,17,18]. Recent publications [9,10,11,12] also covered the spectroscopic characteristics of Ce^3+^ ions in garnets with Ca^2+^, Mg^2+^, and Si^4+^ dopants in the respective {A}, [B], and (C) positions of the garnet host. According to [9], YAG:Ce displayed a lower threshold of thermal quenching of the Ce^3+^ luminescence than that of the Ca_3_Sc_2_Si_3_O_12_:Ce garnet.

Nonetheless, the impact of the simultaneous Mg^2+^–Si^4+^ pair codoping on the optical characteristics of a single Ce^3+^-doped YAG crystal has not been investigated in detail. This is mainly connected with difficulties in the crystallization of a solid Y_3_Mg_x_Si_y_Al_5−x−y_O_12_:Ce solution with conventional growth methods such as the Czochralski or micropulling down techniques. A good answer to this issue is the liquid-phase epitaxy (LPE) technique. The LPE method enables receiving a wide variety of optical materials in single crystalline film form with an extremely low concentration of host defects for the basic research of the optical properties of these materials, and the creation of various luminescent materials on their base for various applications such as laser media [19,20,21], scintillators [22,23,24,25], cathodoluminescent [26,27,28] and scintillating screens [29,30,31], thermoluminescent detectors [32,33,34], and WLED converters [35,36].

The first attempts to obtain Ce^3+^-doped Y_3−x_Ca_x_Al_5−x_Si_x_O_12_:Ce, Ca_3_Sc_2_Si_3_O_12_:Ce, and Ca_2_RSc_2_Si_3_O_12_:Ce ({R} = Y, Lu) garnets in SCF form using the LPE technique for the fabrication of optoelectronic components as blue LED converters or scintillators were presented in our previous works [37,38,39,40].

The optical and photovoltaic characteristics of Y_3_Mg_x_Si_y_Al_5−x−y_O_12_:Ce SCFs with the values of x and y varying in the x = 0–0.345 and y = 0–0.31 ranges are examined in this study, along with new systematic results on growth. The LPE technique was used to produce the SCFs of these garnets onto undoped YAG substrates (see also [37,38,39,40]). Meanwhile, LPE growth methods may also be utilized to create the composite film–substrate epitaxial structures of these garnets for high-power WLED converters. Furthermore, considering that only phosphors based on YAG:Ce crystals or ceramics are available for producing high-power WLEDs under blue LED excitations, the development of this new type of phosphor is a very promising trend in solid-state lighting technology [2,3]. At the same time, we consider the rare-earth and transition metals doped of the SCFs of silicate garnet as potential raw materials for developing novel SCF cathodoluminescent screens, scintillators, and photovoltaic devices [7,8,37,38,39,40].

## 2. Growth of Y_3_Mg_x_Si_y_Al_5−x−y_O_12_:Ce Single Crystalline Films

Using the LPE technique, three sets of optically perfect Y_3_Mg_x_Si_y_Al_5−x−y_O_12_:Ce SCF samples with nominal equimolar Mg and Si contents in a melt solution equal to x, y = 1, 1.5, and 2 were grown onto YAG substrates with a (110) orientation from the supercooling melt–solution (MS) based on the PbO–B_2_O_3_ flux (Table 1). Additionally, another set of samples with nominal Mg and Mg–Si contents in MS in the 0–0.1 range were grown using the LPE method in order to study the photocurrent characteristics of doped Y_3_Mg_x_Si_y_Al_5−x−y_O_12_:Ce SCFs (Table 1). The initial components for LPE growth were PbO, B_2_O_3_, Y_2_O_3_, Al_2_O_3_, SiO_2_, and CeO_2_ oxides of 4N purity.

The SCF contents of the SCF samples were measured using an EDX detector with a SEM JEOL JSM-820 electron microscope. The measurements were performed at five different points of the samples, and the results are averaged. The contents of the Y_3_Mg_x_Si_y_Al_5−x−y_O_12_:Ce SCFs under study and the reference YAG:Ce SCF sample are presented in Table 1.

**Figure 1:**
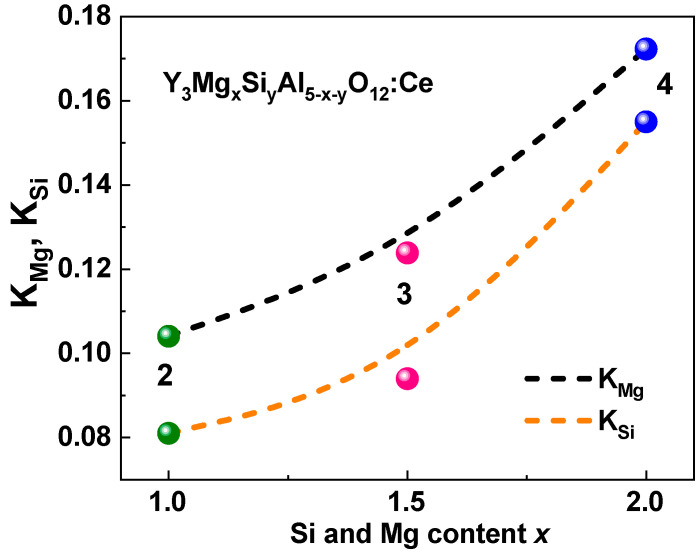
Dependence of Mg and Si segregation coefficients in LPE-grown Y_3_Mg_x_Si_y_Al_5−x−y_O_12_:Ce SCFs (Samples 2–4), while the nominal Mg and Si contents in MS changed in the x = 1–2 range.

The segregation coefficients of Mg^2+^ and Si^4+^ ions in Y_3_Mg_x_Si_y_Al_5−x−y_O_12_:Ce SCFs were defined from the microanalytical measurement of the compositions of these SCF samples grown at the nominal Mg(x) and Si(y) contents of these cations in the corresponding MS (Figure 1). Significant changes in the segregation coefficients of Mg and Si ions in the LPE growth of Y_3_Mg_x_Si_y_Al_5−x−y_O_12_:Ce SCFs were caused by variations in the ratio of Mg/Si/Al cations in the MS (Figure 1). In particular, when the nominal Mg and Si contents in the MS increased in the x = 1–2 range, the segregation coefficients of the Mg^2+^ and Si^4+^ ions in as-grown films were nonlinearly changed in the 0.08–0.155 and 0.105–0.17 ranges, respectively. As a result, the real Mg and Si ion amounts in the Y_3_Mg_x_Si_y_Al_5−x−y_O_12_:Ce SCF samples were varied correspondingly in the x = 0.104–0.345 and y = 0.081–0.31 ranges, respectively. Following the change in SCF growth temperature, the segregation coefficient of Ce^3+^ ions in the above-mentioned garnet hosts changed from 0.017 to 0.025. As a result, the Ce content in the SCF samples was only in the range of 0.175–0.225 at. % at the average Ce concentration in the MS of around 10 mole %.

The real concentrations of Mg and Si in SCFs are not equal, even at equimolar amounts of these ions in the MS, especially at the low contents of these dopants (Table 1). The Mg^2+^ concentration was systematically higher than the Si^4+^ content, as Table 1 demonstrates. This indicates that, for the local charge compensation of Mg^2+^ excess, various 4+ ion states can be formed, for instance, Ce^4+^ ions or Pb^4+^ flux-related ions. The local charge compensation of Mg^2+^ excess can also occur through the formation of O^−^ centers or oxygen vacancies [41,41]. Regarding Y_3_Mg_x_Si_y_Al_5−x−y_O_12_ SCFs, we could predict the presence of both forms of charge compensation of Mg^2+^ excess: the dominant creation of the oxygen vacancies or O^2−^ centers at relatively low Mg^2+^–Si^4+^ contents, and the preferential formation of Ce^4+^ and Pb^4+^ states at relatively high Mg–Si amounts (Figure 1).

**Figure 2:**
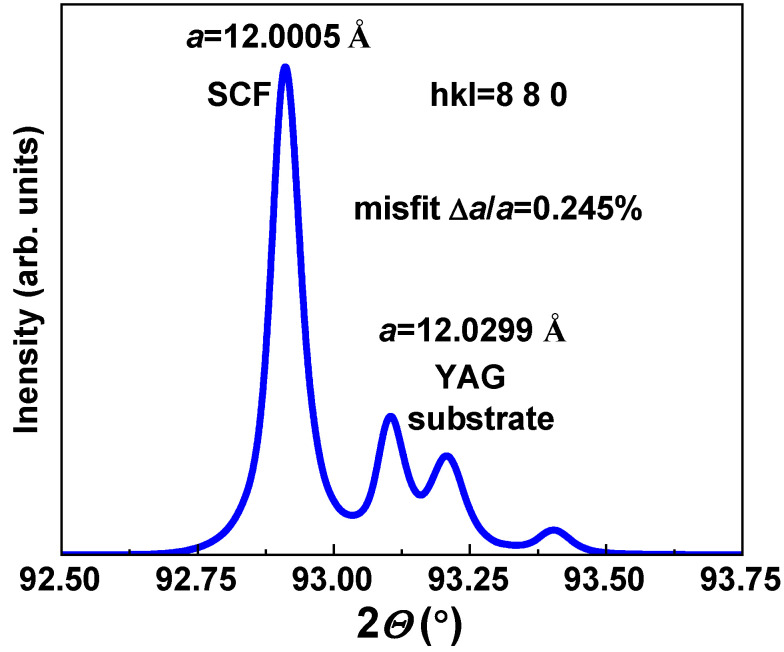
XRD pattern (880) of Y_2.96_Ce_0.04_Mg_0.345_Si_0.31_Al_4.345_O_12_ SCF (Sample 4) grown onto a YAG substrate. The lattice mismatch of the film and substrate was 0.245%.

The structural quality of Y_3_Mg_x_ Si_y_Al_5−x−y_O_12_:Ce SCFs with varying Mg and Si contents, grown using the LPE method onto YAG substrates with (110) orientation with a lattice constant of 11.9930 Ȧ, was characterized using XRD measurements, performed using a modified DRON 4 spectrometer (Cu_Kα_ radiation) (Figure 2). The mismatch between the lattice constants of SCF and YAG substrates as **Δa = (a_SCF_ – a_sub_)/a_sub_** × 100% being equal to 0.245% was evaluated from the respective XRD patterns of the SCF sample grown from an MS with a nominal Y_3_Mg_2_Si_2_Al_3_O_12_:Ce composition and real Y_2.96_Ce_0.04_Mg_0.345_Si_0.31_Al_4.345_O_12_ content (Figure 2). Additionally, we estimated that the lattice constant of the mentioned garnet composition from the XRD pattern was equal to 12.0224 Ȧ.

## 3. Experimental Methods and Technique

**Figure 3:**
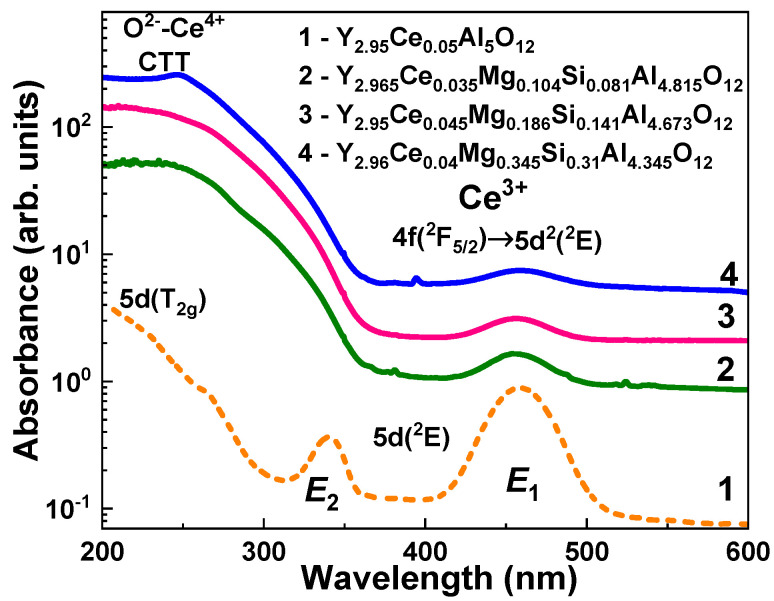
RT absorbance spectra of Y_3_Mg_x_Si_y_Al_5−x−y_O_12_:Ce samples (the curves correspond to the samples with numbers in accordance with Table 1). SCFs with various Mg and Si contents in the x = 0.104–0.345 and y = 0.081–0.31 ranges (Curves 2–4) compared to the CL spectra of the YAG:Ce SCF (Curve 1).

**Figure 4:**
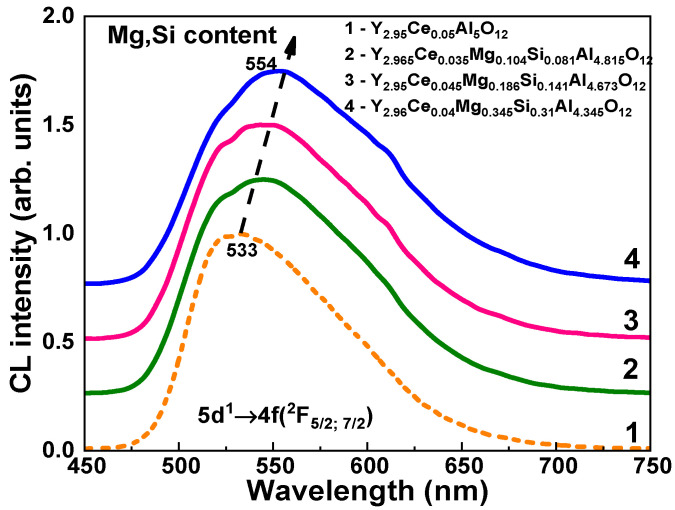
Normalized to the maximal emission band RT CL spectra of the Y_3_Mg_x_Si_y_Al_5−x−y_O_12_:Ce SCFs with various Mg and Si contents in the x = 0.104–0.345 and y = 0.081–0.31 ranges (Curves 2–4) compared to the CL spectra of the YAG:Ce SCF (Curve 1).

**Figure 5:**
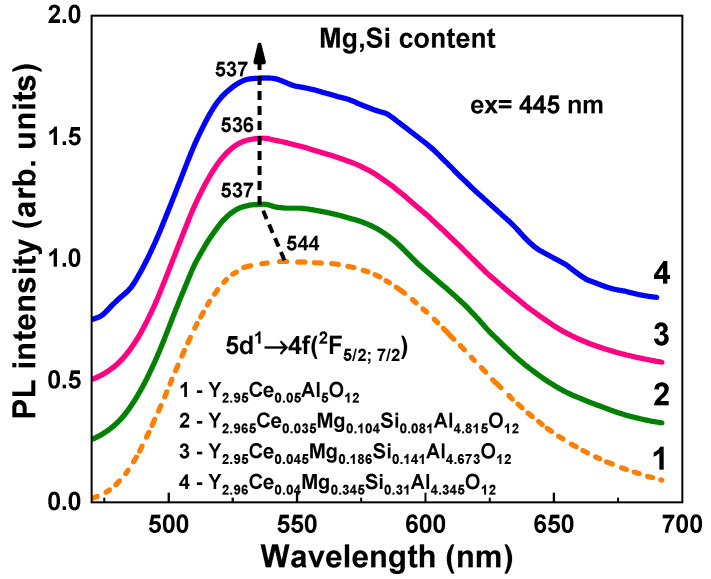
Normalized (to the maximal emission band) RT PL spectra of Y_3_Mg_x_Si_y_Al_5−x−y_O_12_:Ce SCFs (Curves 2–4) with varying Mg and Si contents (Curves 2–4) in comparison to the PL spectra of YAG:Ce SCF (Curve 1) under excitation in the range of the Ce^3+^ absorption band at 445 nm.

**Figure 6:**
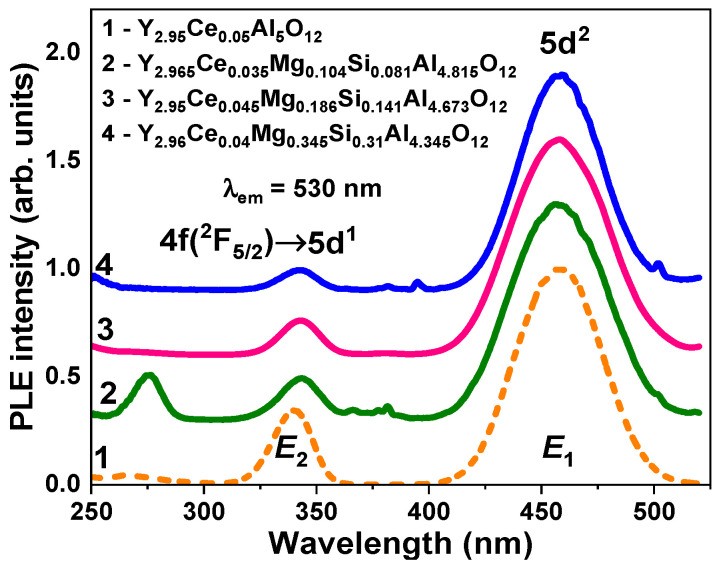
Normalized (to the maximum of the excitation band) RT PLE spectra of Ce^3+^ ion luminescence at 530 nm in Y_3_Mg_x_Si_x_Al_5−x−y_O_12_:Ce SCFs with varying Mg and Si contents (Curves 2–4) compared to the respective PLE spectra in the YAG:Ce SCF (Curve 1).

The absorption (Figure 3), cathodoluminescence (CL) (Figure 4), photoluminescence (PL) (Figure 5), and PL excitation (PLE) (Figure 6) spectra, and the PL decay kinetics (Figure 8 and Table 2) were recorded to characterize the optical and luminescence properties of Y_3_Mg_x_ Si_y_Al_5−x−y_O_12_:Ce SCFs. We also measured the scintillation decay kinetics and photoelectron light yield (LY) of these SCF samples under excitation with α-particles from a ^239^Pu (5.15 MeV) source (Table 3 and Figure 9). The photocurrent (PC) excitation spectra of especially prepared Y_3_Mg_x_ Si_y_Al_5−x−y_O_12_:Ce SCFs (Samples 5–8 in Table 1)) with reduced nominal Mg^2+^ and Mg^2+^–Si^4+^ contents in the x, y = 0–0.1 range were investigated on a custom setup consisting of a 150 W xenon lamp (LOT Quantum Design) coupled to a grating monochromator (Omni-λ 1509) operating in the 250–1000 nm spectral range as a source of excitation; a digital electrometer (Keysight B2987A) for photocurrent measurement; an optical chopper at 5 Hz to modulate the excitation light to increase the signal-to-noise ratio; a lock-in amplifier (Signal Recovery 7270, Ametek Scientific Instruments) to extract the photocurrent signal.

A SEM JEOL JSM-820 electron microscope with a Stellar Net grating spectrometer operating in the 200–1120 nm spectral range was used to measure the CL spectra. An Edinburgh FS5 spectrometer was used to study the PL emission and excitation spectra, and PL decay kinetics of the SCF samples. Using a Hamamatsu H6521 PMP, multichannel analyzer, and digital TDS3052 oscilloscope setup, the scintillation LY with a shaping time of 12 s and decay kinetics were measured under excitation with α-particles of ^239^Pu (5.15 MeV) source. Furthermore, the absorption, luminescence, scintillation, and PC properties of the Y_3_Mg_x_Si_y_ Al_5−x−y_O_12_:Ce SCFs were compared with the properties of the reference YAG:Ce SCF sample. All PC and luminescence measurements were carried out at room temperature (RT).

## 4. Absorption, Luminescence, and Photoconductivity Properties of Y_3_Mg_x_Si_y_Al_5−x−y_O_12_:Ce Films

### 4.1. Absorption Spectra

Figure 3 shows the absorption spectra of Y_3_Mg_x_Si_y_Al_5−x−y_O_12_:Ce SCFs with various Mg/Si contents in comparison with the spectra of the YAG:Ce SCF. The detected E_1_ and E_2_ absorption bands peaked at 458.5 and 340 nm, respectively, corresponding to the 4f^1^(^2^F_5/2_) → 5d (T_2g_) transitions of Ce^3+^ ions (Figure 3, curve 1). The 4f^1^(^2^F_5/2_) → 5d (T_2g_) transitions of Ce^3+^ ions are responsible for the bumps at 230 nm in the spectra of these SCFs. The maximal positions of the E_1_ band in the Y_3_Mg_x_Si_y_Al_5−x−y_O_12_:Ce SCFs with different Mg/Si amounts were slightly shifted in the 456–459 nm range compared to the maximum of the respective band at 458.5 nm in YAG:Ce SCF (Figure 3, Curves 2–4).

In addition to these bands, another wide band that peaked at about 248 nm was present in the absorption spectra of the Y_3_Mg_x_Si_y_Al_5−x−y_O_12_:Ce SCFs. This band strongly overlapped with the E_2_ band (Figure 3, Curves 2–4). The position of this band was close to the identical transitions in Ca^2+^ and Mg^2+^ codoped crystals, and the single crystalline films of Lu_3_Al_5_O_12_:Ce and Gd_3_Ga_3_Al_2_O_12_:Ce and Lu_3_Al_5_O_12_:Ce garnets [42,43,44,45,46,47,48,49]. This suggests that the nature of this band may be related to the O^2−^ → Ce^4+^ charge transfer transitions (CTT) [42,43,44,45,46,47,48,49]. Indeed, the Ce^3+^ and Ce^4+^ valence states coexist in the as-grown Y_3_Mg_x_Si_y_Al_5−x−y_O_12_:Ce SCFs, as confirmed by the existence of the respective absorption bands of these centers, as shown in Figure 3. The relative concentrations of these centers were highly influenced by Mg/Si/Ce contents and SCF crystallization conditions. Specifically, the intensity of the Ce^3+^ E_1_ band decreased with increasing Mg^2+^ and Si^4+^ contents in the x = 0.104–0.345 and y = 0.081–0.31 ranges, probably as a result of the Ce^3+^ → Ce^4+^ recharge. The beginning of the O^2−^ → Ce^4+^ CTT in the studied SCFs could even be shifted to 400 nm, causing a large overlap of the E_2_ absorption bands of the Ce^3+^ ions.

In addition to Ce^3+^-associated bands, the absorption spectra of the Y_3_Mg_x_Si_y_Al_5−x−y_O_12_:Ce SCFs grown from PbO-based flux had bands peaking at 260–263 nm. These bands correspond to the ^1^S_0_ → ^3^P_1_ transitions of Pb^2+^ ions as the main flux pollution in the SCFs grown from PbO-based flux [21]. The comparable band in the YAG:Ce SCF analogue peaked at 263 nm (Figure 3, Curve 1).

### 4.2. Cathodoluminescence Spectra

The normalized CL spectra of Y_3_Mg_x_Si_y_Al_5−x−y_O_12_:Ce SCF samples at RT with various Mg/Si contents are presented in Figure 4 (Curves 2–4) in comparison with their YAG:Ce counterpart (curve 1). The dominant luminescence band in the YAG:Ce SCF with the maximum at 533 nm (Figure 4, Curve 1) occurred in this garnet and corresponded to the Ce^3+^ ion’s 5d^1^ → 4f(^2^F_5/2; 7/2_) transitions. With increasing Mg and Si contents, the location of these bands in Y_3_Mg_x_Si_x_Al_5−2x_O_12_:Ce SCFs displayed a strong red shift up to 554 nm (Figure 4, Curves 2–4). In contrast to YAG:Ce SCFs, the CL spectra of Y_2.96_Ce_0.04_Mg_0.345_ Si_0.31_Al_4.345_O_12_ SCF were actually red-shifted by 21 nm. Furthermore, the Ce^3+^ emission bands in Y_3_Mg_x_Si_y_Al_5−x−y_O_12_:Ce SCFs were noticeably wider than those in YAG:Ce. Namely, in Y_2.96_Ce_0.04_ Mg_0.345_Si_0.31_Al_4.345_O_12_, the respective FWHM value of the Ce^3+^ emission band was equal to 0.466 eV, but in the YAG:Ce SCF, it was only 0.396 eV (Figure 4).

### 4.3. Photoluminescence Spectra

Under stimulation in the vicinity of the *E_1_* Ce^3+^ absorption band at 445 nm, the wide PL band in Y_3_Mg_x_Si_y_Al_5−x−y_O_12_:Ce SCFs peaked at 550 nm for the Y_2.95_Ce_0.05_Al_5_O_12_ and at approximately 536 nm for other SCFs with varied Mg/Si contents. This PL band corresponded to the radiative 5d^1^ → 4f(^2^F_5/2,7/2_) transitions of Ce^3+^ ions (Figure 5). The position of the PL emission bands and their FHWM in Y_3_Mg_x_Si_y_Al_5−x−y_O_12_:Ce SCFs under 450 nm excitation, however, reveal a more complex dependency on the x and y values than that indicated by the CL spectra (Figure 4). In particular, the PL spectra of Y_3_Mg_x_Si_y_Al_5−x−y_O_12_:Ce SCFs had a considerable blue shift relative to the spectra of YAG:Ce SCF, which was only 7–8 nm (Figure 5). Furthermore, the PL spectra of these SCFs were notably narrower than the YAG:Ce counterpart. Namely, for the Y_2.965_Ce_0.045_Mg_0.186_Si_0.141_Al_4.763_O_12_ and Y_2.96_Ce_0.04_Mg_0.345_Si_0.31_Al_4.635_O_12_ SCFs, the respective FWHM values were 0.478 and 0.467 eV in comparison with FWHM = 0.495 eV for the YAG:Ce SCF. Therefore, the PL spectra of Y_3_Mg_x_Si_y_Al_5−x−y_O_12_:Ce SCFs showed different trends with changing the Mg^2+^–Si^4+^ concentrations compared with the corresponding CL spectra (Figure 4). This further indicates the complicated nature of the Ce^3+^ center formation in these garnets and the influence of some variables on the process.

Two E_1_ and E_2_ bands with peaks at 460 nm and in the 340–343 nm range, respectively, were observed in the excitation spectra of the Ce^3+^ luminescence in Y_3_Mg_x_Si_y_Al_5−x−y_O_12_:Ce SCFs. These bands were associated with the 4f(^2^F_5/2_) → 5d^1,2^ transitions of Ce^3+^ ions in these garnets (Figure 6). Namely, for Samples 2–4 of the Y_3_Mg_x_Si_y_Al_5−x−y_O_12_:Ce SCFs (Figure 6), difference ΔE = *E*_2_ − *E*_1_, which was proportional to the crystal field strength in the dodecahedral position of the garnet, was equal to 0.894, 0.901, and 0.907 eV, respectively. These ΔE values significantly differed from the ΔE = 0.935 eV value in the YAG:Ce SCF. Additionally, the Stokes shift was much smaller in the Y_3_Mg_x_Si_y_Al_5−x−y_O_12_:Ce SCF samples (Sample 2: 90 nm and 0.448 eV; Sample 3: 81 nm and 0.406 eV; Sample 4: 79 nm and 0.396 eV) in comparison with that in the YAG:Ce SCF (91.5 nm; 0.451 eV).

Even at the significantly large content of Mg^2+^ ions in the 0.31–0.345 range, the bands that peaked at 275 and around 375 nm in the excitation spectra, related to the intrinsic transitions of F^+^ centers [40,50], were not found in the excitation spectra of the Ce^3+^ luminescence in the Y_3_Mg_x_Si_y_Al_5−x−y_O_12_:Ce SCFs (Figure 6). Such results contradict the results of Y_3−x_Ca_x_Si_y_Al_5−y_O_12_:Ce SCFs, where the creation of F^+^ centers was observed for the compensation of the excess of divalent Ca^2+^ ions [40]. However, the results for Y_3_Mg_x_Si_y_Al_5−x−y_O_12_:Ce SCFs correlate well with the investigation results of the Gd_3_Al_5−x_Ga_x_O_12_:Ce,Mg crystal [41], where the emission of F^+^ was also not found. Therefore, the excess of Mg^2+^ ions in the Y_3_Mg_x_Si_y_Al_5−x−y_O_12_:Ce SCFs was compensated by other mechanisms that are probably connected with the creation of Ce^4+^ states or/and O^2−^–Mg^2+^ pair centers [41].

**Figure 7:**
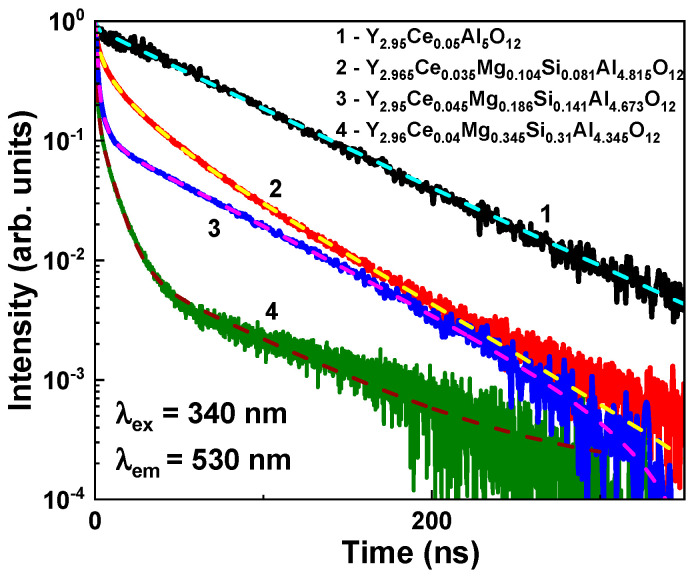
RT decay kinetics of Ce^3+^ luminescence at 530 nm in Y_3_Mg_x_Si_y_Al_5−x−y_O_12_:Ce SCFs with various Mg and Si contents (Curves 2–4) under excitation at 404 nm and registration of PL at 530 nm, compared with the decay kinetics of Ce^3+^ emission in YAG:Ce SCF (curve 1). The respective approximations of the decay curves are presented by the dashed lines.

Figure 7 shows the decay kinetics of the Ce^3+^ ion emission in the Y_3_Mg_x_Si_y_Al_5−x−y_O_12_:Ce SCFs with various Mg and Si contents under excitation at 340 nm near the E_2_ Ce^3+^ ion absorption bands compared to the YAG:Ce SCF counterpart. The decay kinetics of the Y_3_Mg_x_Si_y_Al_5−x−y_O_12_:Ce SCFs (Figure 7, Curves 2–4) was significantly nonexponential in contrast to the YAG:Ce SCF (Figure 7, Curve 1) and similar to that of other A^2+^–Si^4+^ (A = Ca, Mg)-based garnets [37,38,40,43,44]. As the *x* and *y* values rose, the corresponding decay curves became faster and more nonexponential. Due to this fact, the decay curves may have been extrapolated by the three components, each with a decay time value t at intensity decay levels of 1/e, 0.1, and 0.001 (Figure 7). Table 2 lists the corresponding decay times of τ_1/e_, τ_1/10_, and τ_1/100_.

Similarly to the results in [37,38,40,43,44], we assumed that the formation of Ce^4+^ valence states was the primary cause of the nonexponential decay kinetics of the Ce^3+^ luminescence in the as-grown Y_3_Mg_x_Si_y_Al_5−x−y_O_12_:Ce SCFs. The intervalence charge transfer (IVCT) transitions that cause quick nonradiative decay channels could also impact the acceleration of the Ce^3+^ decay in the presence of Ce^4+^ ions [51,52,53]. Recent studies described this effect for Ce^3+^/Ce^4+^ couples in garnets and sulfides [52,53]. Additionally, we recently demonstrated that Ce^4+^ ions, which serve as highly efficient electron trapping centers, may significantly accelerate the decay kinetics of Ce^3+^ luminescence under excitation with the energies in the vicinity of O^2−^ → Ce^4+^ CTTs [38,39,40,41,42,43,44]. The initiation of the O^2−^ → Ce^4+^ transitions is also possible under 340 nm excitation in the E_2_ Ce^3+^ absorption band in garnets due to the substantial FWHM value of the mentioned CTT bands [42,43,44]. The charge transfer of Ce^4+^ into the Ce^3+^ state and the subsequent reverse transformation of Ce^3+^ into Ce^4+^ ions allowed for us to observe the luminescence of Ce^3+^ ions under 340 nm excitation [38,39,40,41,42,43,44].

According to this supposition, the fast components of the cerium luminescence with a lifetime of t_1/e_ = 1.1–13 ns in Y_3_Mg_x_Si_y_Al_5−x−y_O_12_:Ce SCFs under 340 nm excitation may have been caused by Ce^4+^ centers, whereas the slower components, with decay times of t_1/20_ = 10–45 and 30–156 ns, were mostly caused by Ce^3+^ ion radiative transitions. Ce^3+^ luminescence in YAG:Ce SCF had decay time constants of t_1/e_ = 60.5 ns, t_1/20_ = 140 ns, and t_1/100_ = 293 ns (Figure 7, Curve 1).

The existence of the fast component of the Ce^3+^ luminescence in the ns range was interesting, and the nonexponential shape of the decay curves in the garnet compounds containing Ca–Mg–Si ions could have been connected to the formation of Ce^3+^ multicenters [38,39,40]. The energy transfer processes between different Ce^3+^ emitting centers could correspond to such a nonexponential form of the decay curves [38,39]. Nevertheless, the presence of Ce^4+^ centers in the as-grown SCFs substantially masked the contribution of the above-mentioned energy transfer mechanisms to the nonexponential PL decay kinetics of the Ce^3+^ luminescence. Consequently, it was only possible to analyze the impact of the energy transfer mechanisms between Ce^3+^ multicenters after the elimination of Ce^4+^ centers by using the thermal treatment of SCFs in a reducing atmosphere [38].

### 4.4. Scintillation Properties Y_3_Mg_x_Si_y_Al_5−x−y_O_12_:Ce SCFs

Because the majority of Ce^3+^ ions in the as-grown samples had been recharged to the Ce^4+^ state, Mg–Si-codoped SCFs had a low scintillation efficiency. Namely, the as-grown Y_3_Mg_x_Si_y_Al_5−x−y_O_12_:Ce SCFs showed a significantly reduced scintillation LY in comparison with that of the YAG:Ce SCF reference sample, which had a LY of 2600 photons/MeV under α-particle excitation with a ^239^Pu (5.15 MeV) source (Table 3). In general, such scintillation properties of Y_3_Mg_x_Si_y_Al_5−x−y_O_12_:Ce SCFs are similar to those of Y_3−x_Ca_x_Al_5−y_Si_y_O_12_:Ce [40] and Ca_2_YMgScSi_3_O_12_:Ce [37,38,39] SCFs, as well as (Lu,Y)_2_SiO_5_:Ce SCFs [54], where the predominant Ce^4+^ valence state of cerium ions in the SCFs, grown from the PbO-based flux, causes their poor-scintillation light output.

**Figure 8:**
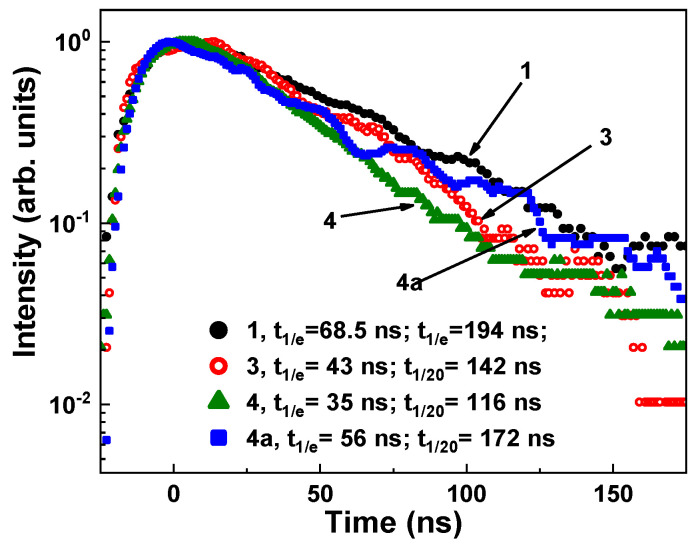
Scintillation decay kinetics of Y_3_Mg_x_Si_y_Al_5−x−y_O_12_:Ce SCF Samples 3 (x/y = 0.186/0.141), 4 (x/y = 0.345/0.31), and 4a in comparison with the respective kinetics of YAG:Ce SCF (Sample 1) under excitation with α-particles using a ^239^Pu (5.15 MeV) source. Sample 4a: the scintillation decay kinetics of SCF Sample 4 after annealing at 1300 °C in an N_2_ 95% + H_2_ 5% reducing atmosphere.

Figure 8 and Table 3 show the scintillation decay kinetics of the Y_3_Mg_x_Si_y_ Al_5−x−y_O_12_:Ce SCFs depending on Mg–Si contents. When Mg–Si concentrations increased, the scintillation response of these SCFs notably accelerate. Namely, for SCF Samples 3 and 4 with Mg/Si contents x/y = 0.186/0.141 and 0.345/0.31, respectively, the corresponding decay times were equal to t_1/e_ = 43 and 35 ns; and t_1/10_ = 142 ns and 116 ns, respectively, in comparison with t_1/e_ = 68.5 ns and t_1/10_ = 194 ns for the YAG:Ce SCF (Table 3). Additionally, this effect was well-correlated with the considerable drop in the LY of Y_3_Mg_x_Si_y_Al_5−x−y_O_12_:Ce SCFs when the Mg/Si content increased (Table 2).

### 4.5. Photocurrent Properties of Y_3_Mg_x_Si_y_Al_5−x−y_O_12_:Ce SCFs

**Figure 9:**
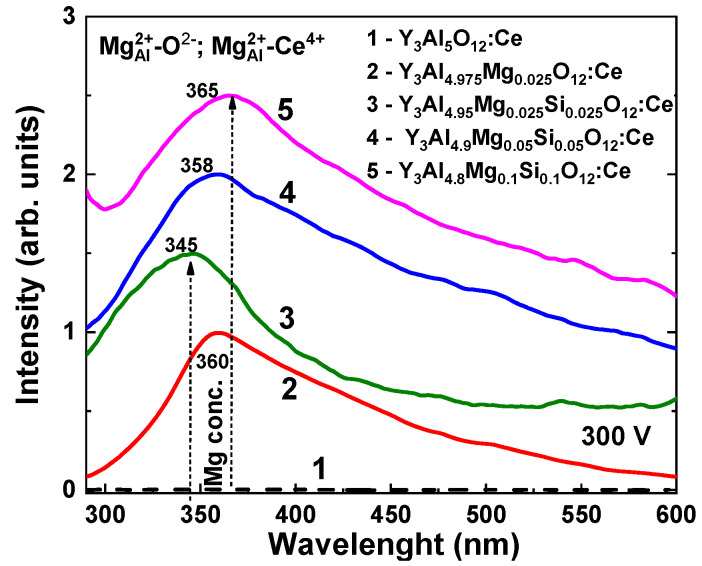
PC excitation spectra of YAG:Ce (Curve 1) and Y_3_Mg_x_Si_y_Al_5−x−y_O_12_:Ce SCFs (Curves 2–5) with different nominal Mg and Si contents x, y = 0.025–0.1 (see Table 1).

The photocurrent (PC) excitation spectra of special set of Y_3_Mg_x_Si_y_Al_5−x−y_O_12_:Ce SCFs with reduced Mg and Si contents between 0.025 and 0.1 for *x* and *y* values are presented in Figure 9. Such reduced amounts of codopants (less than 0.05 at %), substituting the octa- and tetrahedral sites of garnet host, were used to ensure the isolated nature of the substitutional defects. In this way, the photoconductive behavior of the doped crystal was in the isolated donor/acceptor regime, analogously to doped semiconductors. The excessive concentration of donor/acceptor states may lead to the formation of quasiband states, and impairs the photoconductive response of the SCF system.

Since the photocurrent signal of the wide band-gap oxides is extremely weak, the excitation measurements were performed via the modulated light technique and extracted using a lock-in amplifier. For this reason, the absolute value of the photocurrent intensity could not be retained, but it was estimated to be in the 0.1–1 pA range.

The Mg–Si-free YAG:Ce SCF did not show any visible PC under excitation in the 250–600 nm range (Figure 9, Curve 1). However, the single Mg^2+^ and double Mg^2+^–Si^4+^ codoping of YAG:Ce SCF led to the appearance of PCs, and the value of such PCs increases with increasing Mg^2+^ and Si^4+^ concentrations in the films. The maxima of the complex PC excitation band in Y_3_Mg_x_Si_y_Al_5−x−y_O_12_:Ce SCFs were observed in the 345–365 range (Figure 9). Such complex bands consist of at least two low-energy and high-energy sub-bands. Interestingly, the maximum of the complex PC band was slightly shifted from 360 to 345 nm at Mg and Mg–Si concentration x = 0.025, and later shifted to 358 and 365 nm when Mg–Si content increased to x = 0.05 and 0.1, respectively (Figure 9).

Taking into account the advanced Mg^2+^ concentrations in the SCFs under study with respect to the content of Si^4+^ ions and partial compensation of such Mg^2+^ advance by Ce^4+^ formation (Table 1), and the absence of F^+^ and F-related centers formation in these samples, the observed bands in the PC excitation spectra could probably be connected with the creation of Mg^2+^–Ce^4+^ and Mg^2+^–O^2−^ centers with local and charge compensation. The above-mentioned shift in the maxima of PC bands could have been caused by the relative concertation of the mentioned pair centers at different contents of Mg and Si ions.

The mechanism of charge and volume compensation of the Mg^2+^ excess in Y_3_Mg_x_Si_y_Al_5−x−y_O_12_:Ce SCFs was different than that in LPE-grown Y_3−x_Ca_x_Al_5−y_ Si_y_O_12_:Ce SCFs (see [40] for details), where the observed Ca^2+^ advance in the SCF samples was compensated with the Ce^4+^ and F^+^ center formation.

## 5. Optical Properties of Y_3_Mg_x_Si_y_Al_5−x−y_O_12_:Ce SCFs Annealing in Reducing Atmosphere

**Figure 10:**
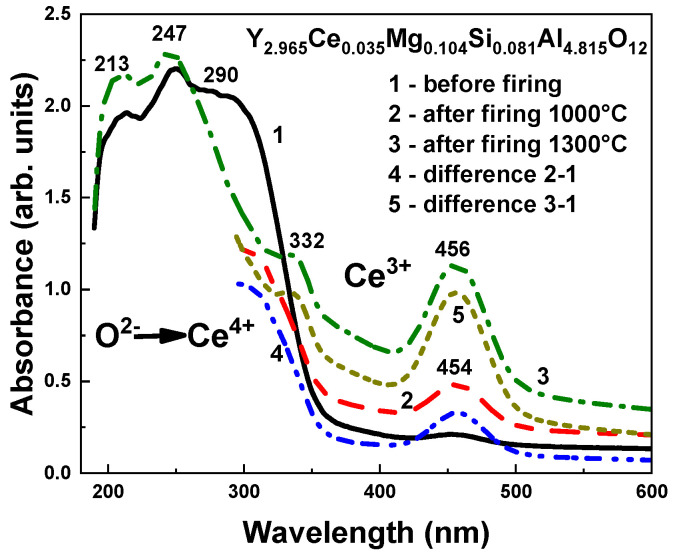
Influence of thermal treatment at 1000 and 1300 °C in a 95% N_2_–5% H_2_ atmosphere on the absorption spectra of the as-grown Y_2.965_Ce_0.035_Mg_0.104_Si_0.081_Al_4.815_O_12_ SCF sample (curve 1). Curves 4 and 5 represent the difference in the spectra of the untreated and annealed samples at 1000 °C (Curve 4) and 1300 °C (Curve 5).

**Figure 11:**
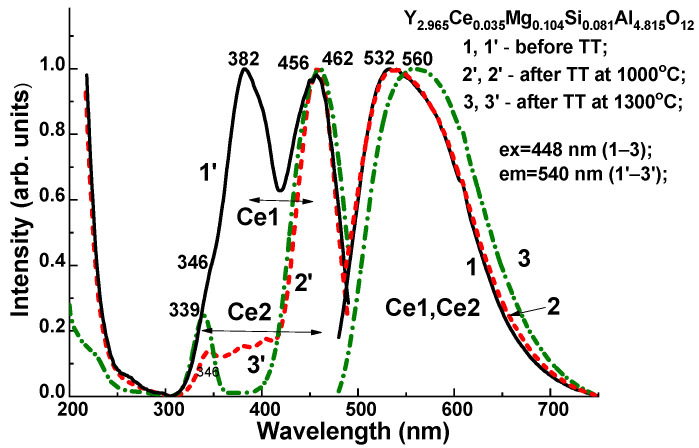
Influence of thermal treatment on the emission spectra (Curves 1–3) and excitation spectra (Curves 1′–3′) of Ce^3+^ luminescence in the Y_2.965_Ce_0.035_Mg_0.104_Si_0.081_Al_4.815_O_12_ SCF in an N_2_ 95% + H_2_ 5% atmosphere at 1300 °C.

**Figure 12:**
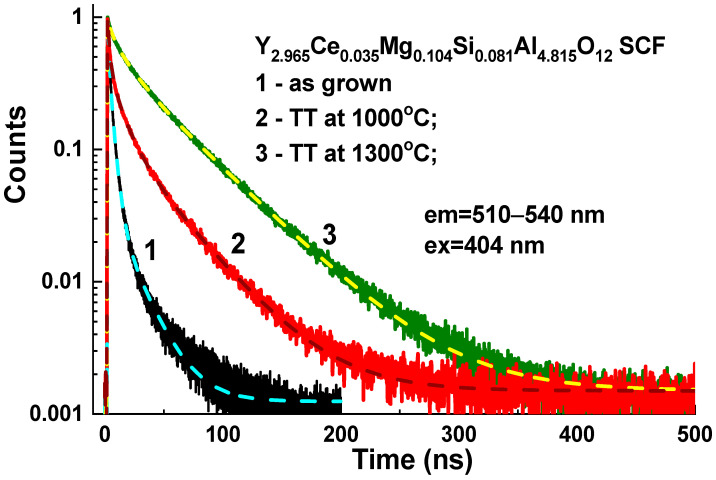
RT decay kinetics of the Ce^3+^ luminescence at 510–545 nm in the Y_2.965_Ce_0.035_Mg_0.104_ Si_0.081_Al_4.815_O_12_ SCF under excitation at 404 nm and registration of PL at 530 nm before (Curve 1) and after TT in reducing N_2_+H_2_ (95 + 5%) atmosphere at 1000 °C (Curve 2) and 1300 °C (Curve 3).

The optical characteristics of Sample 2 (Y_2.965_Ce_0.035_Mg_0.104_Si_0.081_Al_4.815_O_12_ SCF) were also investigated after 12 h of thermal treatment (TT) at 1000–1300 °C in a 95% N_2_–5% H_2_ reducing atmosphere (Figure 10, Figure 11 and Figure 12). The relative concentrations of the Ce^4+^ and Ce^3+^ centers in the above-mentioned SCF sample were significantly altered by the TT in such a reducing atmosphere as a result of the O^2−^ + 2 Ce^4+^ → V_O_ + 2 Ce^3+^ reaction, where Vo is the oxygen vacancy, as can be seen from the absorption spectra of the as-grown and annealed samples of this film in Figure 10.

The structure of the emission and excitation bands connected to various Ce^3+^ centers noticeably change because of annealing of this SCF sample in the reducing atmosphere. In particular, the maximum of the Ce^3+^ emission band was located at 532 nm in the untreated Y_2.965_Ce_0.035_Mg_0.104_ Si_0.081_Al_4.815_O_12_ SCF, and excited in the bands that peaked at 382 and 456 nm (Figure 11, Curve 1). We assumed that the above-described bands may have been connected with the Ce1 center. The difference between the locations of the *E*_1_ and *E*_2_ excitation bands for such a Ce1 center could be equal to 0.526 eV. The Stokes shift was proportional to the difference between the emission and low-energy excitation bands, and for the Ce1 center, it was equivalent to 76 nm (0.388 eV).

However, as a result of the TT of the Y_2.965_Ce_0.035_Mg_0.104_Si_0.081_Al_4.815_O_12_ SCF at 1300 °C, the maximum of the Ce^3+^ emission spectrum was noticeably shifted to 560 nm, the intensity of the excitation band peaked at 382 nm significantly decreases, and a new excitation band appears with a maximum at 339 nm. The mentioned shift in emission and excitation spectra can be attributed to an increase in the relative concentration of Ce2 centers in the SCF sample after TT. The difference between the locations of the E1 and E2 excitation bands for such a Ce2 center was equal to 0.972 eV. Therefore, due to the larger crystal field strength of Ce2 centers than that of Ce1 centers, the position of the emission band of the Ce2 center was red-shifted relative to the Ce1 center. The difference between the positions of the emission and low-energy excitation bands was proportional to the Stokes shift and equal to 98 nm (0.469 eV) for the Ce2 center.

Lastly, we could assume that the Ce1 and Ce2 centers had been formed when Ce^3+^ ions replaced Y^3+^ cations with different local environment caused by the nonuniform distribution of the Mg^2+^ and Si^4+^ cations in the octahedral and tetrahedral position of the garnet host. This assumption about the nature of the Ce1 and Ce2 centers was supported by the corresponding changes in the absorption and PL excitation spectra, and by the PL emission spectra and decay kinetics of PL (Figure 12, Table 4) in the Y_2.965_Ce_0.035_ Mg_0.104_Si_0.081_Al_4.815_O_12_ SCF, which were related to the change in the concentration of the Ce^4+^ and Ce^3+^ centers after reducing TT in the 1000–1300 °C range. Since Ce2 centers in the as-grown samples had the predominant Ce^4+^ valence state, it was difficult to record these centers in the PL emission and excitation spectra, and decay kinetics of the Ce^3+^ luminescence (Figure 3, Figure 4, Figure 5, Figure 6 and Figure 7). However, when the Ce^4+^ ions recharged to the Ce^3+^ states during the TT at temperatures between 1000 and 1300 °C, it was also possible to observe the Ce2 centers in the PL spectra and the decay kinetics of the Ce^3+^ emission (Figure 10, Figure 11 and Figure 12).

## 6. Conclusions

The single crystalline films (SCFs) of Y_3_Mg_x_Si_y_Al_5−x−y_O_12_:Ce garnet at *x* and *y* changing from 0 to 0.345 and 0.31, respectively, were crystallized using the LPE growth method from a melt solution based on the PbO–B_2_O_3_ flux onto Y_3_Al_5_O_12_ (YAG) substrates at the SCF–substrate misfit from 0 up to 0.245%. The segregation coefficients of Mg and Si ions in these SCFs were varied in the 0.08–0.155 and 0.105–0.17 ranges, respectively, when the nominal concentration of these dopants in the melt solution was changed in the x, y = 0–2 range. Additionally, Mg^2+^ excess was systematically present in the as-grown Y_3_Mg_x_Si_y_Al_5−x−y_O_12_:Ce SCFs, which was presumable compensated by the Ce^4+^ ion and Mg^2+^–O^2−^ center formation. Especially prepared Mg^2+^/Mg^2+^–Si^4+^ codoped YAG:Ce SCFs with low concentrations of manganese and silicon ions also demonstrated the appearance of a photocurrent that increased with rising Mg^2+^ and Si^4+^ contents in the films.

The absorption and luminescence properties of Y_3_Mg_x_Si_y_Al_5−x−y_O_12_:Ce SCFs were studied and compared with the properties of the reference YAG:Ce SCF sample. As a result of the Mg^2+^–Si^4+^ pair codoping, the cathodoluminescence spectra of Ce^3+^ ions in the Y_3_Mg_x_Si_y_Al_5−x−y_O_12_:Ce SCFs were noticeably extended in the red range compared to those of the YAG:Ce SCFs due to the Ce^3+^ multicenter formation in the dodecahedral sites of the lattice of the mentioned mixed garnets. Furthermore, we confirmed the formation of two types of Ce^3+^ centers of Y_3_Mg_x_Si_y_Al_5−x−y_O_12_:Ce in the emission and excitation spectra of the Ce^3+^ photoluminescence in the SCFs of these garnets. These two centers (Ce1 and Ce2) possessed various local surroundings due to replacement with the Mg^2+^ and Si^4+^ ions of Al^3+^ cations in the octahedral and tetrahedral sites of the garnet host and were characterized by differing spectral behaviors.

The as-grown Y_3_Mg_x_Si_y_Al_5−x−y_O_12_:Ce SCF samples exhibited poor scintillation properties. Under α–particle excitation through the ^239^Pu (5.15 MeV) source, these SCFs had a fast scintillation response with decay times in the t_1/e_ = 30–43.5 ns and t_1/20_ = 79–148 ns ranges, but a relative low light yield (LY) of 14–19% in comparison with the reference YAG:Ce SCF. However, the LY of Y_3_Mg_x_Si_y_Al_5−x−y_O_12_:Ce SCFs could increase after their annealing in a reducing atmosphere (95% N_2_ + 5% H_2_) at a temperature above the SCF growth temperature.

The simultaneous formation of the Ce^4+^ and Ce^3+^ valence states was also observed in the Y_3_Mg_x_Si_y_Al_5−x−y_O_12_:Ce SCFs due to the nonuniform distribution of the Mg^2+^ and Si^4+^ cations and charge compensation requirement. The presence of Ce^4+^ ions in the as-grown SCFs was confirmed via the presence of the O^2−^ → Ce^4+^ absorption band that peaked at 247 nm. The Ce^4+^ centers were also responsible for the acceleration of the initial stage of the cerium photoluminescence decay profiles, and for the presence of fast components with a lifetime in the range of a few ns in these SCFs. The annealing of the samples in the reducing atmosphere at temperatures over 1000 °C resulted in the Ce^4+^ → Ce^3+^ recharge in the Y_3_Mg_x_Si_y_Al_5−x−y_O_12_:Ce SCFs, and also led to the more exponential-like decay kinetics of the Ce^3+^ luminescence in these SCFs. This allows for studying the energy transfer processes between different Ce^3+^ centers in this garnet.

## Figures and Tables

**Table 1:** Nominal (in MS) and actual (in film) compositions of Y_3_Mg_x_Si_y_Al_5−x−y_O_12_:Ce and YAG:Ce SCFs, LPE grown onto YAG substrates from MS based on the PbO–B_2_O_3_ flux. n. m., not measured.

Samples	Nominal Film Content in MS	Real SCF Content
1	Y_3_Al_5_O_12_:Ce	Y_2.95_Ce_0.05_Al_5_O_12_
2	Y_3_MgSiAl_4_O_12_:Ce	Y_2.965_Ce_0.035_Mg_0.104_Si_0.081_Al_4.815_O_12_
3	Y_3_Mg_1.5_Si_1.5_Al_2_O_12_:Ce	Y_2.95_Ce_0.045_Mg_0.186_Si_0.141_Al_4.673_O_12_
4	Y_3_Mg_2_Si_2_AlO_12_:Ce	Y_2.96_Ce_0.04_Mg_0.345_Si_0.31_Al_4.345_O_12_
5	Y_3_Mg_0.025_Al_4.975_O_12_:Ce	n.m.
6	Y_3_Mg_0.025_Si_0.025_Al_4.95_O_12_:Ce	n.m.
7	Y_3_Mg_0.05_Si_0.05_Al_4.9_O_12_:Ce	n.m.
8	Y_3_Mg_0.1_Si_0.1_Al_4.8_O_12_:Ce	Y_2.965_Ce_0.045_Mg_0.01_Si_0.01_Al_4.98_O_12_

**Table 2:** LY and decay times of the PL at RT in Y_3_Mg_x_Si_y_Al_5−x−y_O_12_:Ce SCFs with various Mg and Si contents under 404 nm excitation and 530 nm PL registration. Decay times were calculated from the parameters of three exponential approximations of the decay curves presented in Figure 7. The accuracy of decay time parameter determination was about ±5%.

No	LY, %	t_1_, ns	A_1_	t_2_, ns	A_2_	t_3_, ns	A_3_
1	100					67.82	0.99
2	19	3.86	0.23	17.89	0.24	52.81	0.18
3	17	1.69	0.32	9.01	0.14	95.09	0.11
4	14	1.85	0.16	6.24	0.27	60.74	0.12

**Table 3:** The decay time of RT PL in Y_3_Mg_x_Si_y_Al_5−x−y_O_12_:Ce SCFs with varying Mg and Si contents under 404 nm excitation and 530 nm PL registration.

No	Real SCF Content	LY, %	t_1/e,_ ns	t_1/10,_ ns
1	Y_2.95_Ce_0.05_Al_5_O_12_	100	68.5	194
3	Y_2.95_Ce_0.045_Mg_0.186_Si_0.141_Al_4.673_O_12_	19	43	142
4	Y_2.96_Ce_0.04_Mg_0.345_Si_0.31_Al_4.345_O_12_	14	35	116
4a	Y_2.96_Ce_0.04_Mg_0.345_Si_0.31_Al_4.345_O_12_	42	56	172

**Table 4:** Parameters of three exponential approximations of the decay curves presented in Figure 12. The accuracy of the decay time parameter determination was about ±5%.

No.	t_1_, ns	A_1_	t_2_, ns	A_2_	t_3_, ns	A_3_
1	3.79	0.53	11.09	0.92	19.61	0.14
2	3.61	0.27	16.03	0.27	50.71	0.47
3	2.47	0.25	12.47	0.23	40.58	0.16

## Data Availability

Not applicable.

## References

[1] Bardsley N., Bland S., Pattison L., Pattison M., Stober K., Welsh F., Yamada M. (2014). Solid-State Lighting R&D. Multi-Year Program Plan.

[2] Sun C.C., Chang Y.Y., Yang T.H., Chung T.Y., Chen C.C., Lee T.X., Li D.R., Lu C.Y., Ting Z.Y., Glorieux B. (2014). Packaging efficiency in phosphor-converted white LEDs and its impact to the limit of luminous efficacy. J. Solid State Light..

[3] Raukas M., Kelso J., Zheng Y., Bergenek K., Eisert D., Linkov A., Jermann F. (2013). Ceramic phosphors for light conversion in LEDs. ECS J. Solid. State Sci. Technol..

[4] Cantore M., Pfaff N., Farrell R.M., Speck J.S., Nakamura S., DenBaars S.P. (2015). High luminous flux from single crystal phosphor-converted laser-based white lighting system. J. Opt. Exp..

[5] Nizhankovskyi S.V., Tan’ko A.V., Savvin Y.N., Krivonogov S.I., Budnikov A.T., Voloshin A.V. (2016). Single crystalline YAG:Ce phosphor for powerful solid-state sources of white light. The influence of production conditions on luminescence properties and lighting characteristics. J. Opt. Spectrosc..

[6] Li S., Zhu Q., Tang D., Liu X., Ouyang G., Cao L., Hirosaki N., Nishimura T., Huang Z., Xie R.J. (2016). Al_2_O_3_:YAG: Ce composite phosphor ceramic: A thermally robust and efficient color converter for solid state laser lighting. J. Mater. Chem. C.

[7] Markovskyi A., Gorbenko V., Zorenko T., Nizhankovskiy S., Fedorov A., Zorenko Y. (2021). Composite color converters based on Tb_3_Al_5_O_12_:Ce single crystalline films and Y_3_Al_5_O_12_:Ce crystal substrates. J. Phys. Status Solidi-Rapid Res. Lett..

[8] Markovskyi A., Gorbenko V., Nizhankovskiy S., Zorenko T., Pakuła M., Kaczmarek M., Fedorov A., Zorenko Y. (2022). Novel composite color converters based on Tb_1.5_Gd_1.5_Al_5_O_12_:Ce single crystalline films and Y_3_Al_5_O_12_:Ce crystal substrates. CrystEngComm.

[9] Setlur A.A., Heward W.J., Gao Y., Srivastava A.M., Chandran R.G., Shankar M.V. (2006). Crystal Chemistry and Luminescence of Ce^3+^-Doped Lu_2_CaMg_2_(Si,Ge)_3_O_12_ and Its Use in LED Based Lighting. J. Chem. Mater..

[10] Shimomura Y., Honma T., Shigeiwa M., Akai T., Okamoto K., Kijima N. (2007). Sensors and Displays: Principles, Materials, and Processing-Photoluminescence and Crystal Structure of Green-Emitting Ca_3_Sc_2_Si_3_O_12_: Ce^3+^ Phosphor for White Light Emitting Diodes. J. Electrochem. Soc..

[11] Katelnikovas A., Bettentrup H., Uhlich D., Sakirzanovas S., Jüstel T., Kareiva A. (2009). Synthesis and optical properties of Ce^3+^-doped Y_3_Mg_2_AlSi_2_O_12_ phosphors. J. Lumin..

[12] Kishore M.S., Kumar N.P., Chandran R.G., Setlur A.A. (2010). Solid Solution Formation and Ce^3+^ Luminescence in Silicate Garnets. Electrochem. Solid-State Lett..

[13] Zhong J., Zhuang W., Xing X., Liu R., Li Y., Liu Y., Hu Y. (2014). Synthesis, Crystal Structures, and Photoluminescence Properties of Ce^3+^-Doped Ca_2_LaZr_2_Ga_3_O_12_: New Garnet Green-Emitting Phosphors for White LEDs. J. Phys. Chem..

[14] Pan Z., Xu Y., Hu Q., Li W., Zhou H., Zheng Y. (2015). Combination cation substitution tuning of yellow-orange emitting phosphor Mg_2_Y_2_Al_2_Si_2_O_12_:Ce^3+^. J. RSC Adv..

[15] Li G., Tian Y., Zhao Y., Lin J. (2015). Recent progress in luminescence tuning of Ce^3+^ and Eu^2+^-activated phosphors for pc-WLEDs. J. Chem. Soc. Rev..

[16] Shang M., Fan J., Lian H., Zhang Y., Geng D., Lin J. (2014). A double substitution of Mg^2+^–Si^4+^/Ge^4+^ for Al (1)3+–Al (2)3+ in Ce3+-doped garnet phosphor for white LEDs. J. Inorg. Chem..

[17] Du Y., Shao C., Dong Y., Yang Q. (2016). Electroluminescent properties of WLEDs with the structures of Ce:YAG single crystal/blue chip and Sr_2_Si_5_N_8_:Eu^2+^/Ce:YAG single crystal/blue chip. J. Disp. Technol..

[18] Zhao B.Y., Liang X., Chen Z., Xie C., Luo L., Zhang Z., Xiang W. (2014). Studies on optical properties and Ce concentration of Ce: YAG single crystal for WLEDs. Chem. J. Chin. Univ..

[19] Ferrand B., Chambazand B., Couchaud M. (1999). Liquid phase epitaxy: A versatile technique for the development of miniature optical components in single crystal dielectric media. J. Opt. Mater..

[20] Molva E. (1999). Microchip lasers and their applications in optical microsystems. J. Opt. Mater..

[21] Klimczak M., Malinowski M., Sarnecki J., Piramidowicz R.J. (2009). Luminescence properties in the visible of Dy:YAG/YAG planar waveguides. J. Lumin..

[22] Zorenko Y., Novosad S.S., Pashkovskii M.V., Lyskovich A.B., Savitskii V.G., Batenchuk M.M., Malyutenkov P.S., Patsagan N.I., Nazar I.V., Gorbenko V.I. (1990). Epitaxial structures of garnets as scintillation detectors of ionizing radiation. J. Appl. Spectrosc..

[23] Zorenko Y., Gorbenko V., Konstankevych I., Grinevand B., Globus M. (2002). Scintillation properties of Lu_3_Al_5_O_12_:Ce single-crystalline films. J. Nucl. Instrum. Methods Phys. Res..

[24] Witkiewicz-Lukaszek S., Gorbenko V., Zorenko T., Syrotych Y., Mares J.A., Nikl M., Sidletskiy O., Bilski P., Yoshikawa A., Zorenko Y. (2022). Composite detectors based on single crystalline films and single crystals of garnet compounds. J. Mater..

[25] Prusa P., Kucera M., Mares J.A., Hanus M., Beitlerova A., Onderisinova Z., Nikl M. (2013). Scintillation properties of the Ce-doped multicomponent garnet epitaxial films. Opt. Mater..

[26] Robertson J.M., Van Tol M.V. (1984). Cathodoluminescent garnet layers. J. Thin Solid Film.

[27] Hrytskiv Z.D., Zorenko Y., Gorbenko V., Pedanand A.D., Shkliarsyi V.I. (2007). Single crystalline film screens for cathode-ray tubes: New life of television scanning optical microscopy. J. Radiat. Meas..

[28] Schauer P., Lalinský O., Kucera M. (2021). Overview of S(T)EM electron detectors with garnet scintillators: Some potentials and limits. J. Microsc. Res. Tech..

[29] Koch A., Raven C., Spanne P., Snigirev A. (1998). X-ray imaging with submicrometer resolution employing transparent luminescent screens. J. Opt. Soc. Amer. A Opt..

[30] Martin T., Koch A. (2006). Recent developments in X-ray imaging with micrometer spatial resolution. J. Synchrotron Radiat..

[31] Riva F., Douissard P.-A., Martin T., Carla F., Zorenko Y., Dujardin C. (2016). Epitaxial growth of gadolinium and lutetium-based aluminum perovskites thin film for X-rays micro-imaging applications. CrystEngComm.

[32] Zorenko Y., Gorbenko V., Savchyn V., Fedorov A., Kuklinski B., Grinberg M., Bilski P., Gieszczyk W., Twardak A., Mandowski A. (2012). Luminescent properties of YAlO_3_:Mn single crystalline films. J. Opt. Mater..

[33] Witkiewicz-Lukaszek S., Gorbenko V., Zorenko T., Zorenko Y., Gieszczyk W., Mrozik A., Bilski P. (2019). Composite thermoluminescent detectors based on the Ce^3+^ doped LuAG/YAG and YAG/LuAG epitaxial structures. J. Radiat. Meas..

[34] Witkiewicz-Lukaszek S., Gorbenko V., Bilski P., Mrozik A., Zorenko T., Fedorov A., Zorenko Y. (2020). LPE growth of composite thermoluminescent detectors based on the Lu_3-x_Gd_x_Al_5_O_12_:Ce single crystalline films and YAG:Ce crystals. J. Cryst..

[35] Markovskyi A., Gorbenko V., Zorenko T., Yokosawa T., Will J., Spiecker E., Batentschuk M., Elia J., Fedorov A., Zorenko Y. (2021). LPE growth of Tb_3_Al_5_O_12_:Ce single crystalline film converters for WLED application. CrystEngComm.

[36] Markovsky A., Gorbenko V., Yokosawa T., Will J., Spiecker E., Batentschuk M., Elia J., Fedorov A., Pakuła M., Kaczmarek M. (2022). Structural, luminescence and photoconversion properties of Lu_3_Al_5_O_12_:Ce single crystalline film phosphors for WLED application. J. Alloy. Compd..

[37] Gorbenko V., Zorenko T., Paprocki K., Iskaliyeva A., Fedorov A., Schröppel F., Levchuk I., Osvet A., Batentschuk M., Zorenko Y. (2017). Epitaxial growth of single crystalline film phosphors based on the Ce^3+-^doped Ca_2_YMgScSi_3_O_12_ garnet. CrystEngComm.

[38] Gorbenko V., Zorenko T., Pawlowski P., Iskaliyeva A., Paprocki K., Suchocki A., Zhydachevskii Y., Fedorov A., Khaidukov N., Van Deun R. (2018). Luminescent and scintillation properties of Ce^3+^ doped Ca_2_RMgScSi_3_O_12_ (R=Y, Lu) single crystalline films. J. Lumin..

[39] Gorbenko V., Zorenko T., Witkiewicz S., Paprocki K., Iskaliyeva A., Kaczmarek A.M., Van Deun R., Khaidukov M.N., Batentschuk M., Zorenko Y. (2018). Luminescence of Ce^3+^ multicenters in Ca^2+^-Mg^2+^-Si^4+^ based garnet phosphors. J. Lumin..

[40] Gorbenko V., Zorenko T., Witkiewicz-Łukaszek S., Shakhno A., Osvet A., Batentschuk M., Fedorov A., Zorenko Y. (2021). Crystallization and investigation of the structural and optical properties of Ce^3+^ doped Y_3-x_Ca_x_Al_5-y_Si_y_O_12_ single crystalline film phosphors. J. Cryst..

[41] Bartosiewicz K., Markovskyi A., Horiai T., Szymański D., Kurosawa S., Yamaji A., Yoshikawa A., Zorenko Y. (2022). A study of Mg^2+^ ions effect on atoms segregation, defects formation, luminescence and scintillation properties in Ce^3+^ doped Gd_3_Al_2_Ga_3_O_12_ single crystals. J. Alloy. Compd..

[42] Wu Y., Meng F., Li Q., Koschan M., Melcher C.L. (2014). Role of Ce^4+^ in the Scintillation Mechanism of Codoped Gd_3_Ga_3_Al_2_O_12_∶Ce. Phys. Rev. Appl..

[43] Tyagi M., Meng F., Koschan M., Donnald S.B., Rothfuss H., Melcher C.L. (2013). Effect of codoping on scintillation and optical properties of a Ce-doped Gd_3_Ga_3_Al_2_O_12_ scintillator. J. Phys. D Appl. Phys..

[44] Liu S., Feng X., Zhou Z., Nikl M., Shi Y., Pan Y. (2013). Effect of Mg^2+^ co-doping on the scintillation performance of LuAG:Ce ceramics. Phys. Status Solidi (RRL)-Rapid Res. Lett..

[45] Babin V., Herman P., Kucera M., Nikl M., Zazubovich S. (2019). Effect of Mg^2+^ co-doping on the photo- and thermally stimulated luminescence of the (Lu,Gd)_3_(Ga,Al)_5_O_12_:Ce epitaxial films. J. Lumin..

[46] Lalinsky O., Schauer P., Kucera M. (2019). Influence of Mg-to-Ce Concentration Ratio on Cathodoluminescence in LuAG and LuGAGG Single-Crystalline Films. Phys. Status Solidi A.

[47] Prusa P., Kučera M., Babin V., Bruza P., Parkman T., Panek D., Beitlerova A., Mares J.A., Hanus M., Lučeničová Z. (2018). Tailoring and Optimization of LuAG:Ce Epitaxial Film Scintillation Properties by Mg Co-Doping. Cryst. Growth Des..

[48] Schauer P., Lalinský O., Kučera M., Lučeničová Z., Hanuš M. (2017). Effect of Mg co-doping on cathodoluminescence properties of LuGAGG:Ce single crystalline garnet films. Opt. Mater..

[49] Babin V., Boháček P., Jurek K., Kučera M., Nikl M., Zazubovich S. (2017). Dependence of Ce^3+^—Related photo- and thermally stimulated luminescence characteristics on Mg^2+^ content in single crystals and epitaxial films of Gd_3_(Ga,Al)_5_O_12_:Ce,Mg. Opt. Mater..

[50] Zorenko Y., Zorenko T., Voznyak T., Mandowski A., Xia Q., Batentschuk M., Fridrich J. (2010). Luminescence of F^+^ and F centers in Al_2_O_3_-Y_2_O_3_ oxide compounds. IOP Conf. Ser. Mater. Sci. Eng..

[51] Barandiarán Z., Meijerink A., Seijo L. (2015). Configuration coordinate energy level diagrams of intervalence and metal-to-metal charge transfer states of dopant pairs in solids. Phys. Chem. Chem. Phys..

[52] Kulesza D., Cybińska J., Seijo L., Barandiarán Z., Zych E. (2015). Anomalous red and infrared luminescence of Ce^3+^ ions in SrS: Ce sintered ceramics. J. Phys. Chem. C.

[53] Phung Q.M., Barandiarán Z., Seijo L. (2015). Structural relaxation effects on the lowest 4f–5d transition of Ce^3+^ in garnets. J. Theor. Chem. Acc..

[54] Zorenko Y., Gorbenko V., Savchyn V., Zorenko T., Grinyov B., Sidletskiy O., Fedorov A. (2014). Growth and luminescent properties of Ce and Ce–Tb doped (Y,Lu,Gd)_2_SiO_5_:Ce single crystalline films. J. Cryst. Growth.

